# Impact of the Hypoechogenicity Criteria on Thyroid Nodule Malignancy Risk Stratification Performance by Different TIRADS Systems

**DOI:** 10.3390/cancers13215581

**Published:** 2021-11-08

**Authors:** Nina Malika Popova, Maija Radzina, Peteris Prieditis, Mara Liepa, Madara Rauda, Kaspars Stepanovs

**Affiliations:** 1 Institute of Diagnostic Radiology, Pauls Stradins Clinical University Hospital, 1002 Riga, Latvia; peteris.prieditis@stradini.lv (P.P.); mara.liepa@rsu.lv (M.L.); madara.rauda@stradini.lv (M.R.); kstepanovs@gmail.com (K.S.); 2 Faculty of Medicine, University of Latvia, 1004 Riga, Latvia; 3 Radiology Research Laboratory, Riga Stradins University, 1002 Riga, Latvia

**Keywords:** TIRADS, thyroid nodule, ultrasound, fine-needle aspiration biopsy

## Abstract

**Simple Summary:**

This study is aimed at raising the question of the use of several TIRADS systems that stratify the risk of thyroid nodule malignancy. Approximately 5–20% of thyroid nodules are malignant, but most nodules are benign, and they are scored by FNA biopsy. One of the goals is to reduce the number of unnecessary FNA and the associated with-it possible complications for the patient and financial cost. Most TIRADS systems are based on the fact that one suspicious feature of a thyroid nodule classifies it as malignant, but there is a modified Kwak et al. system that is based on the count of malignant features. Therefore, this study is intended to estimate the specificity, sensitivity, and accuracy of the systems and, in the future, think about reducing the number of FNA biopsies. The result of this study can be important for all doctors who face thyroid changes, such as radiologists, ultrasonography specialists, and endocrinologists, those who must decide about the need for an FNA.

**Abstract:**

Background: Various Thyroid Imaging and Reporting data systems (TIRADS) are used worldwide for risk stratification of thyroid nodules. Their sensitivity is high, while the specificity is suboptimal. The aim of the study was to compare several TIRADS systems and evaluate the effect of hypoechogenicity as a sign of risk of malignancy on the overall assessment of diagnostic accuracy. Methods: The prospective study includes 274 patients with 289 thyroid nodules to whom US and risk of malignancy were assessed according to four TIRADS systems—European (EU-TIRADS), Korean (K-TIRADS), TIRADS by American College of Radiology (ACR TIRADS), and modified Kwak et al. TIRADS (L-TIRADS) systems, in which mild hypoechogenicity is not included in malignancy risk suggestive signs. For all thyroid nodules, a fine needle aspiration (FNA) biopsy was performed and evaluated according to the Bethesda system. For all systems, diagnostic accuracy was calculated. Results: Assessing the echogenicity of the thyroid nodules: from 81 of isoechogenic nodules, 2 were malignant (2.1%), from 151 mild hypoechogenic, 18 (12%) were malignant, and from 48 marked hypoechogenic nodules, 16 (33%) were malignant. In 80 thyroid nodules, mild hypoechogenicity was the only sign of malignancy and none appeared malignant. Assessing various TIRADS systems on the same cohort, sensitivity, specificity, PPV, NPV, and accuracy, firstly for EU-TIRADS, they were 97.2%; 39.9%; 18.7%; 99.0%, and 73.3%, respectively; for K-TIRADS they were 97.2%; 46.6%; 20.6%; 99.2%, and 53.9%; for ACR-TIRADS they were 97.2%; 41.1%, 19.0%; 99.0%, and 48.0%, respectively; finally, for L-TIRADS they were 80.6%; 72.7%; 29.6%; 96.3%, and 73.3%. Conclusions: This comparative research has highlighted that applying different TIRADS systems can alter the number of necessary biopsies by re-categorization of the thyroid nodules. The main pattern that affected differences was inconsistent hypoechogenicity interpretation, giving the accuracy superiority to the systems that raise the malignancy risk with marked hypoechogenicity, at the same time with minor compensation for sensitivity.

## 1. Introduction

The thyroid nodules are very common in the general population, and with the increase of imaging techniques, more thyroid nodules are accidentally detected [1]. Comparing the clinical examination with ultrasonographic examination, it was found that 46% of nodules (with a diameter >1 cm) detected with ultrasound (US) were not detected during the physical thyroid examination [2,3]. Therefore, nowadays the ultrasound (US) is the primary diagnostic method for patients with thyroid nodules. However, with the implementation of ultrasonography in the diagnosis of thyroid nodules, overdiagnosis and overtreatment occurs, which entails an increase in surgical operations and possible complications, as well as financial costs for the treatment of thyroid replacement therapy.

Nodular thyroid disease is relatively common. Most of the thyroid nodules are benign and asymptomatic, and their prevalence varies and accounts for about 85–93% in the population; in addition, 20% of them decrease in size over a lifetime [4]. About 80% of nodular thyroid diseases are caused by glandular hyperplasia, which occurs in up to 5% of the population. Its etiology includes iodine deficiency (endemic), hormonal diseases (congenital family forms) and poor iodine absorption because of certain medications [2]. Calcifications, which are often rough and perinodular, can occur during cyst degeneration. Pure cystic formation is rarely cancerous, but malignancy probability in the nodules with solid and cystic components reaches the prevalence of cancer in solid nodules [5].

Adenomas account for only 5% to 10% of all nodular thyroid diseases. In most cases, thyroid dysfunction is not observed, and less than 10% have hyperfunction, which can lead to thyrotoxicosis. Usually adenomas are solitary, but they can also develop as part of a multinodular formation [2].

Benign follicular adenoma is a true neoplasm characterized by compression of adjacent tissues and fibrous encapsulation. Cytologically, the features of follicular adenoma are usually indistinguishable from those of follicular carcinoma. Signs of follicular carcinoma are vascular and capsule infestations that can be detected during histology, so fine needle aspiration (FNA) is not considered as a reliable method to distinguish adenoma from carcinoma; therefore, tumors are usually removed surgically [6].

However, thyroid cancer is rare and accounts for less than 1% of all malignancies [4]. It accounts for 5% to 10% of all thyroid nodules and there are differentiated thyroid carcinomas, i.e., papillary, follicular, Hürthle cell carcinomas, as well as medullary and anaplastic thyroid carcinomas. Papillary thyroid carcinoma is the most common malignancy of the thyroid gland and accounts for about 80–85% of all thyroid cancers [7]. However, papillary carcinoma has an excellent prognosis with a 10-year survival rate of about 90% [8]. There are many histological subtypes of papillary carcinoma, but ultrasound features that are suggestive of this type of cancer are nodule hypoechogenicity, microlobulated or spiculated margins, microcalcifications in the structure, and taller-than-wide orientation [9].

Follicular carcinoma is the second most common thyroid cancer type and accounts for 10 to 15% of all thyroid cancer [10]. The challenge for the ultrasound specialist is to differentiate follicular carcinoma from follicular adenoma, but ultrasonographic features such as large, hypoechogenic nodule, lack of halo and absence of cystic component are more associated with follicular carcinoma than with adenoma [11].

Medullary thyroid carcinoma accounts for 3.5 to 10% of all thyroid cancers and is characterized by early lymphatic metastatic spread, aggressive invasion in adjacent tissues and organs, and has a poor prognosis. On the ultrasound examination, medullary carcinoma usually looks like papillary carcinoma and is perceived as a solid, hypoechogenic nodule, often with calcifications, which may look more visually coarser than in papillary carcinoma. Calcifications are observed in the primary tumor as well as in the metastatic lymph nodes [6].

Anaplastic thyroid carcinoma usually affects elderly people and is one of the deadliest solid thyroids tumors. It accounts for less than 2% of all thyroid cancers but has the worst prognosis, with 5-year mortality above 95%. The tumor manifests with a rapidly growing mass that extends beyond the gland and grows into adjacent structures. It does not tend to lymphogenic dissemination, but it aggressively grows in adjacent muscles and fat tissue. Sonographically, anaplastic cancer is usually hypoechogenic and often surrounds or grows into blood vessels and neck muscles. Often, these tumors cannot be properly evaluated by the ultrasound due to their large size. In this case, the extent of damage can be assessed more accurately by computed tomography or magnetic resonance imaging [6].

In this regard, the clinical challenge is to distinguish clinically significant malignant thyroid nodules and thus to identify patients who will require surgery for benign thyroid nodules and who will require long-term observation. That is why the main purpose of US thyroid nodule examination is to differentiate malignant nodules from benign [12,13,14].

In the past decade, several professional societies and research groups have implemented guidelines to provide a standardized assessment of ultrasonographic features of thyroid nodules, such as Thyroid Imaging Reporting and Data System (TIRADS), to assess the need for fine needle aspiration biopsy (FNA) [15].

Initially, in 2009, Horvath et al. introduced a thyroid malignancy risk stratification system—TIRADS [16], but it was difficult to implement, and as a result, several national thyroid associations introduced their own thyroid assessment models—American College of Radiology, European and Korean TIRADS systems.

Most variants of the TIRADS systems include mild hypoechogenic nodules in the potential risk group or TIRADS 4, however, several studies report that most thyroid nodules are mild hypoechogenic, both benign and malignant [17,18,19,20].

Several studies have been performed comparing thyroid malignancy risk assessment systems. The sensitivity of these systems is high enough, but specificity is quite low. Therefore, in this study we compare four TIRADS systems—Kwak et al., hereafter, referred to as L-TIRADS, European TIRADS (EU-TIRADS), Korean TIRADS (K-TIRADS) and American College of Radiology TIRADS (ACR-TIRADS) systems—including the sensitivity and specificity and evaluate the effect of hypoechogenicity as a sign of risk of malignancy on the overall assessment of diagnostic accuracy.

## 2. Materials and Methods

### 2.1. Study Design

This was a prospective multicentre study that was conducted in the period from 2019 to 2021. In total, 274 patients with 289 thyroid nodules underwent the conventional ultrasound (US) examination and all patients with clinical indication for fine needle aspiration biopsy (FNA) were included in the study. The study was approved by the institutional review boards and the responsible ethics committee.

### 2.2. Acquisition of Data

Without a regular clinical description of ultrasonographic examination, a standard protocol was additionally developed, with included patient data, suspicious ultrasound features of thyroid nodules, as well as all the necessary features for such systems as the TIRADS system used in Latvia (L-TIRADS), European (EU-TIRADS), Korean (K-TIRADS), and ACR TIRADS system. In addition, the protocol included such indicators as multinodular goiter, thyroiditis, suspicious lymph nodes in four groups of the neck—lateral and medial lymph node groups of right and left side.

### 2.3. US Examination

In the study, thyroid ultrasonography was performed with an ultrasonography device “Canon Aplio i800” with a linear probe i18LX5 (5–18 MHz), and with “Philips EPIQ 5G” with a linear probe eL 18-4. The ultrasound examination was performed by one of five certified radiologists with five and more years of experience. All ultrasound and biopsies, as well as documentation, were carried out by one radiologist. After ultrasound, the description documented the localization of the thyroid nodules, its size, contour, borders, shape, internal components, echogenicity, calcifications, vascularization, as well as the appearance of the thyroid parenchyma and lymph nodes of the neck.

### 2.4. Qualification of Thyroid Nodules by Modified Kwak et al. TIRADS, European TIRADS, Korean TIRADS, and ACR TIRADS Systems

Based on ultrasonographic features, each thyroid nodule was evaluated according to four TIRADS systems.

**Figure 1:**
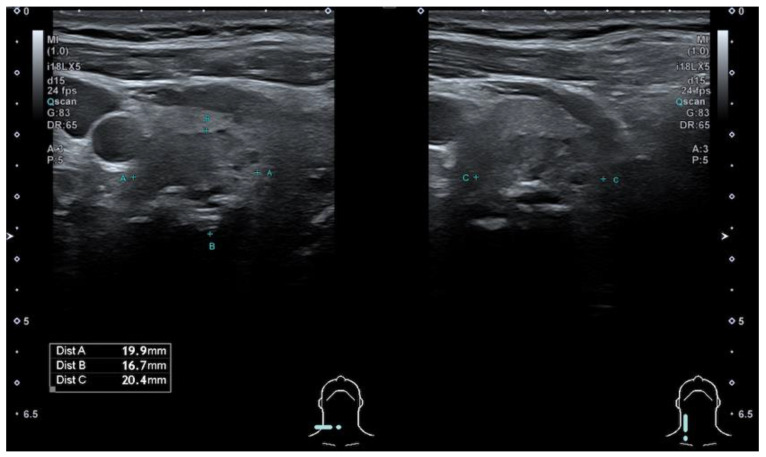
Hypoechogenic, ovoid, smooth, non-homogeneous solid thyroid nodule with cystic component and macrocalcification in its structure—categorized as TIRADS 3 by modified Kwak et al. (L-TIRADS), TIRADS 4 by European and Korean TIRADS, TIRADS 5 (5 points) by ACR TIRADS systems. FNA results—Bethesda 2 (benign).

**Figure 2:**
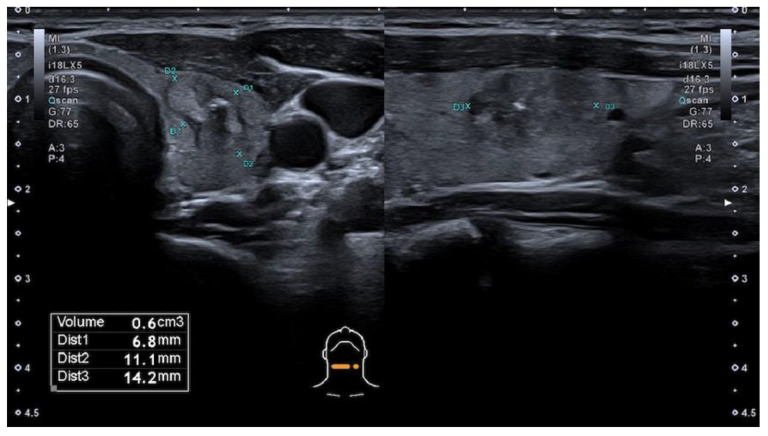
Hypoechogenic nodule, ovoid shape, smooth margins, non-homogeneous solid thyroid nodule with cystic component and macrocalcification in its structure—TIRADS 3 by modified Kwak et al. (L-TIRADS), TIRADS 4 by European and Korean TIRADS, TIRADS 5 by ACR TIRADS systems. FNA biopsy results—Bethesda 2.

The modified Kwak et al. TIRADS system [21] that is used in Latvia (L-TIRADS) is based on the count of suspicious signs of ultrasound, which include marked hypoechogenicity, microcalcifications, taller than wide shape, irregular or microlobulated/ spiculated margins and metastatic lymph nodes. TIRADS 1—normal thyroid tissue without nodules. TIRADS 2 includes simple cysts, spongiform nodules, hyperechogenic nodules in patients with chronic autoimmune thyroiditis; multinodular hyperplastic goiter without separate bounded nodules; isolated macrocalcifications. TIRADS 3 includes partly cystic nodules; solid nodules with isoechogenic, hyperechogenic, moderately hypoechogenic structure, without any independent sign of malignancy (Figure 1 and Figure 2). TIRADS 4A—one ultrasonographic sign of malignancy, 4B—two ultrasonographic signs of malignancy, 4C—three ultrasonographic signs of malignancy, and 5—four and more ultrasonographic signs of malignancy.

According to the European TIRADS system [22] (EU-TIRADS), thyroid nodules were classified as EU-TIRADS 2 if it was a simple cyst or entirely spongiform nodule, EU-TIRADS 3—ovoid, smooth isoechogenic/ hyperechogenic nodule without high suspicion features, TIRADS 4—ovoid, smooth, mild hypoechogenic nodules without high suspicion features, and thyroid nodules were classified as TIRADS 5, if at least one of the following features of high suspicion were included—marked hypoechogenicity, irregular shape, and margins, microcalcifications, with the presence of solid component.

In accordance with the Korean TIRADS system [23] (K-TIRADS), thyroid nodules were classified as K-TIRADS 2 if it was a simple cyst, partially cystic nodule with comet tail artifact, or spongiform nodule. Partially cystic, iso/hyperechoic nodules without any suspicion features were classified as TIRADS 3, and with any suspicious US features such as microcalcification, non-parallel orientation, or spiculated/microlobulated margin such as TIRADS 4. Furthermore, as TIRADS 4 were classified thyroid nodules if they were solid, hypoechogenic without any suspicion US features. TIRADS 5 were classified as solid, hypoechogenic nodules with any suspicious US features—non-parallel orientation, spiculated/ microlobulated margin, and microcalcification in nodule structure.

American College of Radiology TIRADS (ACR TIRADS) [24] is based on points throughout the following categories: composition, echogenicity, shape, margin, and echogenic foci. Composition (select 1): cystic or almost completely cystic—0 points, spongiform—0 points, mixed cystic and solid—1 point, solid or almost completely solid—2 points. Echogenicity (select 1): anechogenic—0 points, hyperechogenic or isoechogenic—1 point, hypoechogenic—2 points, very hypoechogenic—3 points. Shape (select 1): wider-than-tall—0 points, taller-than-wide—3 points. Margin (select 1): smooth—0 points, ill-defined—0 points, lobulated or irregular—2 points, extrathyroidal extension—3 points. Echogenic foci (select all that apply): none or large comet-tail artifacts—0 points, macrocalcifications—1 point, peripheral (rim) calcifications—2 points, punctate echogenic foci—3 points. To determine ACR TIRADS level points from all categories need to be added—ACR-TIRADS 1—0 points, ACR-TIRADS 2—2 points, ACR-TIRADS 3—3 points, ACR-TIRADS 4—4 to 6 points, ACR-TIRADS 5—7 points and more.

### 2.5. Fine Needle Aspiration Biopsy (FNA)

The US-guided FNA biopsy was performed with a 23-gauge needle, three aspirations were performed according to the local standards, and the material was applied onto glass slides and sent for cytology evaluation [25,26]. Samples were analyzed by certified histopathologists with experience in thyroid pathologies, and thyroid nodule FNA materials were classified into Bethesda classification categories: I—non-diagnostic or unsatisfactory, II—benign, III—atypia of undetermined significance or follicular lesion of undetermined significance, IV—follicular neoplasm or suspicious of a follicular neoplasm, V—suspicious of malignancy, VI—malignant [27,28].

### 2.6. Statistical Analysis

IBM SPSS software, 22.0 version (IBM Corm., Armonk, NY, USA), MS Excel 2010 software were used to analyse statistical data and create graphs. Pearson correlation was used for correlation between Bethesda categories and all four TIRADS systems that were included in the study. Sensitivity (SEN), specificity (SPE), positive predictive value (PPV), negative predictive value (NPV), accuracy (ACC), 95% confidence interval (CI), area under curve (AUC) were calculated for each TIRADS system—L-TIRADS, EU-TIRADS, K-TIRADS, and ACR-TIRADS. Cronbach’s Alpha was calculated for reliability between L-TIRADS and EU-TIRADS, L-TIRADS and K-TIRADS, L-TIRADS and ACR-TIRADS. The statistical significance level of this study was assumed to be *p*-value < 0.05.

## 3. Results

### 3.1. Characteristics of the Study Population

For a duration of 18 months, 289 thyroid nodule ultrasounds and fine-needle aspirations in 274 patients were analysed. Most patients were women (233; 85.1%), and only 41 (14.9%) men were included in this study. Using the binomial test, it was concluded that in our study the proportion of women and men (85.1%/14.9%) was statistically significantly different (*p* < 0.001). The age of the patients included ranged from 23 to 85 years, with a mean age of 55 years. The mean age was 55 years for women and 52 years for men. Most patients were in the age group from 60 to 69 years—69 patients (23 %).

### 3.2. Characteristics of the Thyroid Nodules

**Figure 3:**
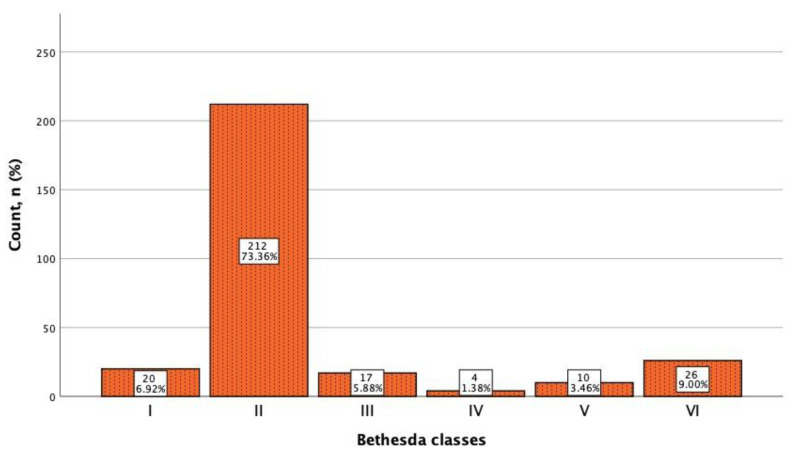
Incidence of cytological findings in thyroid nodules by Bethesda classes.

All 289 thyroid nodules included in the study underwent FNA biopsy, and the resulting cytological material was evaluated according to the Bethesda cytological classification, as shown in Figure 3. All 26 malignant nodules were surgically treated and proved to be papillary carcinomas.

**Figure 4:**
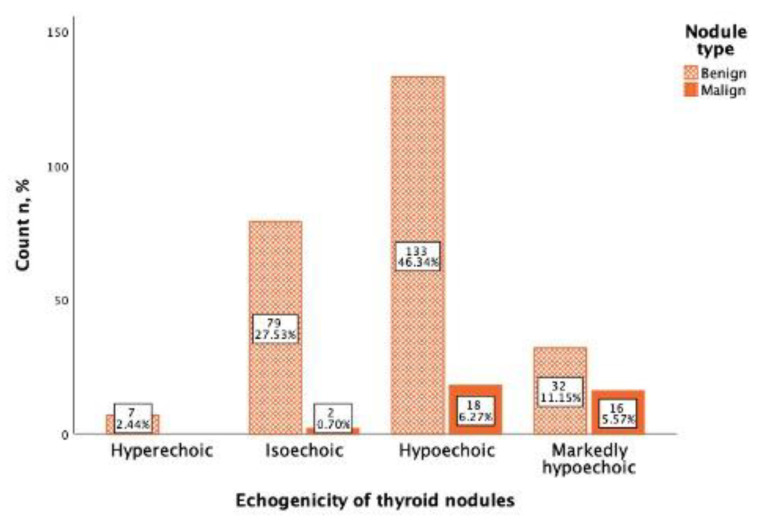
Thyroid nodule malignancy depending on nodule echogenicity. Benign nodules (orange) and malignant nodules (red) are displayed in four ultrasound echogenicity categories.

In the study, 151 (52.6%) thyroid nodules were mild hypoechogenic compared to thyroid parenchyma, of which 133 (88.0%) were benign and 18 (12.0%) were malignant. A total of 48 thyroid nodules were marked hypoechogenic, compared to the neck muscles of which 32 (66.(6)%) were benign and 16 (33.(3)%) were malignant. From 81 (28.2%) isoechoic nodules 79 (97.5%) were benign, and only 2 (2.5%) were malignant, and all hyperechoic nodules (7; 2.4%) were benign (Figure 4). Using Spearman correlation, a negative, statistically significant, weak correlation between Bethesda classification and thyroid nodule echogenicity was found (rs = −0.187, *p* = 0.002).

**Figure 5:**
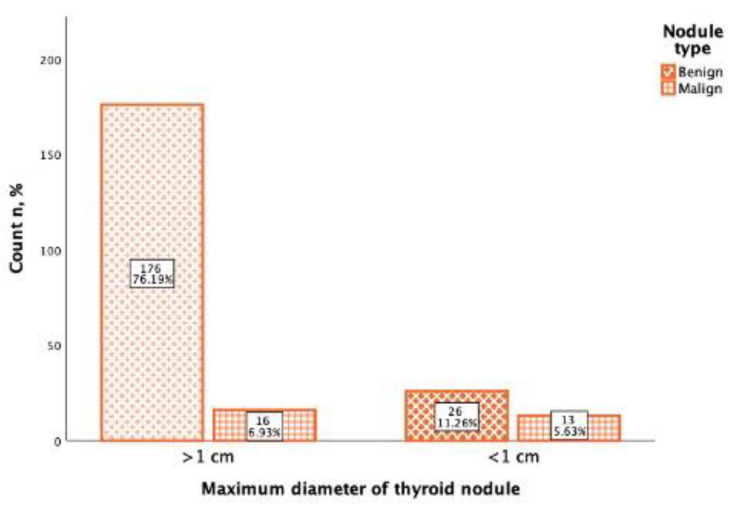
Thyroid nodule malignancy depending on the nodule maximal diameter. Benign nodules are marked with the oblique line pattern and malignant nodules with the check box pattern.

In the study of 231 thyroid nodules, the maximum diameter was noted, from which the majority (192; 83.1%) of thyroid nodules were more than 1 cm diameter (>1 cm), and 39 (16.9%) thyroid nodules were less than 1 cm diameter (<1 cm). From 192 thyroid nodules that were >1 cm diameter, 176 (91.6%) were benign, and 16 (8.4%) were malignant. Of the 39 thyroid nodules that were <1 cm diameter, 26 (66.(6)%) were benign, and 13 (33.(3)%) were malignant (Figure 5). Using Spearman correlation, it was found that there was a positive, statistically significant, weak correlation between the Bethesda classification and the thyroid maximum nodule diameter (rs = 0.283, *p* <0.001).

### 3.3. TIRADS Systems Data Analysis

A summary of thyroid nodules by cytological Bethesda category and L-TIRADS, EU-TIRADS, K-TIRADS, and ACR-TIRADS is shown in Table 1, Table 2, Table 3 and Table 4. Most of the thyroid nodules were in the Bethesda category II or benign 212 (73.4%), although EU-TIRADS, K-TIRADS, ACR-TIRADS categorized them as TIRADS 3 category in 21.1 to 24.6% cases, the L-TIRADS system reached up to 46.4% cases, showing the superiority of the latter system because of mild hypoechogenicity interpretation as a benign sign.

Category I or non-diagnostic results were observed in 20 (6.9%) fine needle aspirations, category III of atypia of undetermined significance were in 17 (5.9%) cases.

IV category or suspicious of follicular neoplasm were in 4 (1.4%) cases, only the L-TIRADS system did not categorized these nodules as TIRADS 5, and the most accurate evaluation was performed by EU-TIRADS and ACR-TIRADS systems.

V category or suspicious of malignancy were in 10 (3.5%) cases, and malignant nodules or category VI were identified in 26 (9.0%) cases. Comparing EU-TIRADS, K-TIRADS, and ACR-TIRADS that showed similar rates of TIRADS 5 nodules in the Bethesda VI category (more than 8%), L-TIRADS categorized the Bethesda VI nodules in TIRADS category 4 A to C, resulting in the reduced sensitivity for TIRADS 5 (2.1%).

Using Pearson correlation, it was found that there was a positive, statistically significant, moderate correlation between Bethesda classification and L-TIRADS (r = 0.509, *p* < 0.001), as well as between Bethesda classification and EU-TIRADS (r = 0.365; *p* < 0.001), between Bethesda classification and K-TIRADS (r = 0.365; *p* < 0.001), and between Bethesda classification and ACR-TIRADS (r = 0.384; *p* < 0.001) systems there was also a positive, statistically significant, moderately strong correlation.

Table 5 compares the TIRADS systems and the number of benign and malignant thyroid nodules according to cytology (Bethesda 5 and 6), depending on the TIRADS category. L-TIRADS showed a markedly increased TIRADS 3 nodules category that was related to the interpretation of mild hypoechogenicity in these nodules in comparison to other systems. There was one malignant thyroid nodule in the TIRADS 3 category in all TIRADS systems. This nodule after check-up was an isoechogenic nodule without any suspicious ultrasound signs.

Table 6 reflects the comparison between TIRADS systems and percentual risk of malignancy of TIRADS categories. L-TIRADS showed lesser malignancy risk in the TIRADS 3 category and higher malignancy risk in TIRADS 5 category in comparison to other TIRADS systems 0.007% and 0.58%, respectively, suggestive of higher diagnostic accuracy in patient selection for FNA.

**Figure 6:**
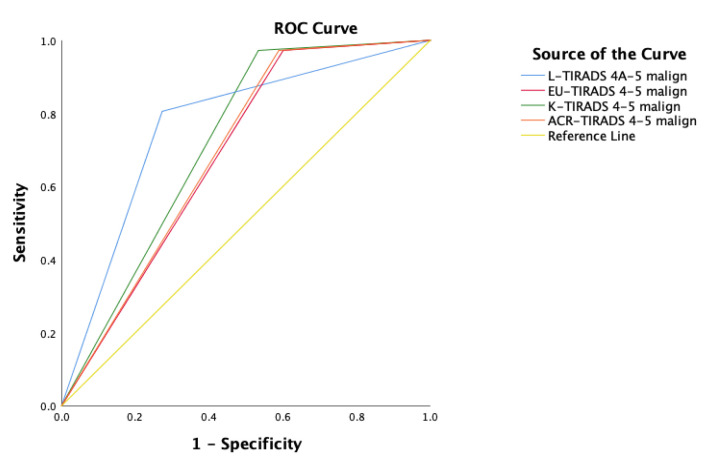
ROC curve analysis for TIRADS systems. L-TIRADS 4A-5 (blue line), EU-TIRADS 5 (red line), K-TIRADS 5 (green line), ACR-TIRADS 5 (orange line), reference line (yellow line).

EU-TIRADS, K-TIRADS, and ACR-TIRADS had high sensitivity (SEN = 97.2%), however L-TIRADS showed higher specificity (SPE = 72.7%) and a better accuracy (ACC = 73.3, *p* < 0.001) than other systems—EU-TIRADS (ACC = 47.0, *p* < 0.001), K-TIRADS (ACC = 53.9, *p* < 0.001) and ACR-TIRADS (ACC = 48.0, *p* < 0.001). All TIRADS systems showed similar area under the ROC curve, but the L-TIRADS AUC value was better 0.766, followed by K-TIRADS with an AUC value 0.719 (Table 7, Figure 6).

In the comparison between L-TIRADS and EU-TIRADS, K-TIRADS, and ACR TI-RADS among the 126 thyroid nodules classified as TIRADS 5 by the EU-TIRADS, 23 were downgraded to 4C, 29 were downgraded to 4B, 57 were downgraded to 4A, 4 were downgraded to TIRADS 3, and 13 were categorized into the same TIRADS category. Among the 82 thyroid nodules classified as TIRADS 4 by the EU-TIRADS, 74 were downgraded to TIRADS 3 by L-TIRADS, and 8 were categorized into the same TIRADS category (Table 8).

A total of 113 thyroid nodules were classified as TIRADS 5 by the K-TIRADS, 23 were downgraded to 4C, 29 were downgraded to 4B, 48 were downgraded to 4A, 3 were downgraded to 3, and 13 were categorized into the same TIRADS category. Out of the 32 thyroid nodules classified as TIRADS 4 by the K-TIRADS, 56 were downgraded to TIRADS 3 by L-TIRADS, and 17 were categorized into the same TIRADS category (Table 9).

In total, 103 thyroid nodules were classified as TIRADS 5 by the ACR-TIRADS, 23 were downgraded to 4C, 28 were downgraded to 4B, 34 were downgraded to 4A, 3 were downgraded to 3, and 12 were categorized into the same TIRADS category. In the group of the 108 thyroid nodules classified as TIRADS 4 by the ACR-TIRADS, 76 were downgraded to TIRADS 3 by L-TIRADS, 31 were categorized as TIRADS 4A, 1 was upgraded to TIRADS 4B, and 1 was upgraded to TIRADS 5 (Table 10).

The reliability between L-TIRADS and all three TIRADS systems with Cronbach’s Alpha was comparable, between L-TIRADS and EU-TIRADS was 0.805, between L-TIRADS and K-TIRADS was 0.817, and between L-TIRADS and ACR-TIRADS was 0.794, respectively.

## 4. Discussion

This prospective comparative study includes ultrasound data from 289 thyroid nodules and fine-needle aspirations from 274 patients, with age ranging from 23 to 85 years (mean 55) and including predominantly women (85.1%), in accordance with the several studies that also noted a higher prevalence of thyroid nodules in women and noted that the incidence of thyroid nodules increases after the age of 50 [29,30]. A total of 26 nodules were proved to be malignant with papillary carcinoma.

Various TIRADS systems (ACR, EU-TIRADS, K-TIRADS, American Thyroid Association etc.) are used to define thyroid nodule risk of malignancy and to select patients for FNA. All systems are based on thyroid nodule malignancy features evaluation such as hypoechogenicity, microcalcifications, shape proportions (AP > LL), ill-defined and irregular margin. For these systems, diagnostic accuracy is similar, with high sensitivity and low specificity [31,32]. In our study, ACR TIRADS sensitivity, specificity, PPV, NPV and diagnostic accuracy were 97.2%; 41.1%, 19.0%; 99.0%, and 48.0%; for K-TIRADS—97.2%; 46.6%; 20.6%; 99.2%, and 53.9%; for EU-TIRADS —97.2%; 39.9%; 18.7%; 99.0%, and 73.3%, respectively, and these results are similar to other authors’ research results [33,34,35].

Hypoechogenicity is one of the main but unspecific features of thyroid nodules on US [36]. The positive predictive value of this feature is weak (PPV and OR ranging from 3.57 to 6.63) [21,37]. In favor of making this pattern more selective, a lower echogenicity compared to muscle tissue termed “marked hypoechogenicity” has been introduced as a marker of increased risk of malignancy in solid nodules. It is reported that up to 55% of benign nodules appear hypoechoic compared to thyroid parenchyma, making nodules not marked as hypoechogenicity less specific, especially for sub-centimeter size [37]. The goal of the present study was to evaluate the diagnostic accuracy of the current TIRADS system. It was hypothesized that in accordance with the assumption of a higher malignancy in markedly hypoechogenic nodules, all remaining hypoechogenic lesions with equal or higher echogenicity compared to muscle tissue (“non-marked hypoechogenic nodules”) would not be associated with an increased risk of malignancy and that their classification as grade 3 would significantly improve the predictive value of the TIRADS classification.

In the L-TIRADS system, which is the Kwak et al. modified version, mild hypoechogenicity is not included in the malignancy features. Mild hypoechogenic thyroid nodules, which do not have any other malignant features, are stratified as thyroid nodules with low risk of malignancy or TIRADS 3 category nodules, which results in higher specificity and diagnostic accuracy, 72.7% and 73.3%, respectively, and therefore showed different performance in comparison to the other systems with remaining high NPV 96.3% and a comprehensive decrease in sensitivity 80.6%. Based on the cytological response, among the nodules categorized as L-TIRADS 5, 58% were cytologically malignant (Bethesda 5 and 6), while from the nodules categorized as K-TIRADS, 5–31% were cytologically malignant, from nodules categorized as EU-TIRADS, 5–29% were cytologically malignant, and from thyroid nodules that were categorized as ACR-TIRADS 5, only 13% were cytologically malignant. Comparing EU-TIRADS, K-TIRADS, and ACR-TIRADS that showed similar rates of TIRADS 5 nodules in the Bethesda VI category (more than 8%), L-TIRADS categorized the Bethesda VI nodules in TIRADS category 4 A to C, resulting in the reduced sensitivity for TIRADS 5 to only 2.1%. This trade-off of the raised suspicion and lack of clear categorization of malignancy is compensated by the L-TIRADS system’s high performance in the TIRADS 3 category with two-times higher true benign nodules (Bethesda 2).

The aim of TIRADS systems is to evaluate thyroid nodule risk of malignancy and to select patients for FNA. In these TIRADS systems, indications for FNA are based on thyroid nodule malignancy risk assessment or TIRADS category and size of the nodule [22,24,35]. Low TIRADS system specificity increases the number of FNA, which results in unnecessary manipulations, stress, and financial costs for the patients.

Analyzing echogenicity as malignancy risk evaluation criteria, it seems that only 12.0% of the mild hypoechogenic nodules were malignant, which means that this feature is not specific by itself, while among the marked hypoechogenic nodules, 66.6% were malignant, which means that every two out of three marked hypoechogenic nodules are malignant and this proves that this feature is specific for malignancy. The result of our study shows that mild hypoechogenicity cannot serve solely as a malignancy risk feature and may increase FNA rates unnecessarily. From 80 thyroid nodules, in which mild hypoechogenicity was the only possible malignancy sign, no cytological malignancy (Bethesda 5 and 6) by FNA was found. This approved the concept of mild hypoechogenicity with lower values to be considered as a sign of malignancy, as described in several TIRADS systems: ACR TI-RADS non-marked hypoechogenicity without other signs is classified as TIRADS 3 (mild suspicious), EU-TIRADS category 4 (intermediate risk) and K-TIRADS also as category 4 (intermediate suspicion) [22,23,24].

Regarding the size of the nodule—one of three nodules with a diameter less than 1 cm was malignant, but it should be noted that most of the small nodules firstly appear as relatively hypoechogenic and for these sub-centimeter nodules, FNA biopsy may be advised only if they have several signs of malignancy.

There were some limitations in our study, such as the examination of the thyroid gland was performed using two ultrasound machines, and in our study, there were several ultrasound specialists who performed examination and FNA, which could lead to different assessments and decisions about FNA of thyroid nodules. Furthermore, some of the patients had to be excluded from the study due to non-informative results of cytology, which may be due to imperfect ultrasound control during FNA, as well as changes in image quality. Lack of histology results in our cohort should be noted as a major limitation.

In conclusion, EU-TIRADS, K-TIRADS, and ACR-TIRADS showed high sensitivity, compared to L-TIRADS; however, the specificity and accuracy were higher for the L-TIRADS system. The main pattern that affected differences was inconsistent hypoechogenicity interpretation, giving accuracy superiority to the systems that raise the malignancy risk with marked hypoechogenicity and at the same time with minor compensation for sensitivity. This comparative research has highlighted that applying different TIRADS systems can alter the number of necessary biopsies by categorization of the mild hypoechogenic thyroid nodules as a low malignancy risk pattern.

## Figures and Tables

**Table 1:** Summary of L-TIRADS categories by Bethesda classes.

		Bethesda Class					
		I	II	III	IV	V	VI
L-TIRADS, *n* (%)	2	-	2 (0.9)	-	-	-	-
	3	12 (4.2)	134 (46.4)	5 (1.7)	1 (0.3)	-	1 (0.3)
	4A	1 (0.3)	53 (18.3)	3 (1.0)	2 (0.7)	5 (1.7)	5 (1.7)
	4B	4 (1.4)	16 (5.5)	2 (0.7)	-	3 (1.0)	4 (1.4)
	4C	2 (0.7)	3 (1.0)	6 (2.1)	1 (0.3)	1 (0.3)	10 (3.5)
	5	1 (0.3)	4 (1.4)	1 (0.3)	-	1 (0.3)	6 (2.1)
Total, *n* (%)		20 (6.9)	212 (73.4)	17 (5.9)	4 (1.4)	10 (3.5)	26 (9.0)

**Table 2:** Summary of EU-TIRADS categories by Bethesda classes.

		Bethesda Class					
		I	II	III	IV	V	VI
EU-TIRADS, *n* (%)	2	-	4 (1.9)	-	-	-	-
	3	5 (1.7)	66 (22.8)	4 (1.4)	1 (0.3)	-	1 (0.3)
	4	7 (2.4)	73 (25.3)	2 (0.7)	-	-	
	5	8 (2.8)	69 (23.9)	11 (3.8)	3 (1.0)	10 (3.5)	25 (8.7)
Total, *n* (%)		20 (6.9)	212 (73.4)	17 (5.7)	4 (1.4)	10 (3.5)	26 (9.0)

**Table 3:** Summary of K-TIRADS categories by Bethesda classes.

		Bethesda Class					
		I	II	III	IV	V	VI
K-TIRADS, *n* (%)	2	1 (0.3)	17 (5.9)	-	-	-	-
	3	6 (2.1)	71 (24.6)	3 (1.0)	1 (0.3)	-	1 (0.3)
	4	5 (1.7)	63 (21.8)	3 (1.0)	1 (0.3)	-	1 (0.3)
	5	8(2.8)	61 (21.1)	11 (3.8)	2 (0.7)	10(3.5)	24 (8.3)
Total, *n* (%)		20 (6.9)	212 (73.4)	17 (5.7)	4 (1.4)	10 (3.5)	26 (9.0)

**Table 4:** Summary of ACR-TIRADS categories by Bethesda classes.

		Bethesda Class					
		I	II	III	IV	V	VI
ACR-TIRADS, *n* (%)	1	1 (0.3)	2 (0.7)	-	-	-	-
	2	-	5 (1.7)	-	-	-	-
	3	6 (2.1)	61 (21.1)	3 (1.0)	1 (0.3)	-	1 (0.3)
	4	5 (1.7)	97 (33.6)	4 (1.4)	-	1 (0.3)	1 (0.3)
	5	8 (2.8)	47 (16.3)	10 (3.5)	3 (1.0)	9 (3.1)	24 (8.3)
Total, *n* (%)		20 (6.9)	212 (73.4)	17 (5.7)	4 (1.4)	10 (3.5)	26 (9.0)

**Table 5:** Comparison between L-TIRADS, EU-TIRADS, K-TIRADS, and ACR-TIRADS categories.

Categories	Based on L-TIRADS	Based on EU-TIRADS	Based on K-TIRADS	Based on ARC-TIRADS
TIRADS 1 benign/malignant	-	-	-	2/0
TIRADS 2 benign/malignant	2/0	4/0	17/0	5/0
TIRADS 3 benign/malignant	140/1	71/1	75/1	65/1
TIRADS 4A * benign/malignant	58/10	75/0	67/1	101/2
TIRADS 4B benign/malignant	18/7	-	-	-
TIRADS 4C benign/malignant	10/7	-	-	-
TIRADS 5 benign/malignant	5/7	83/35	74/34	233/36

* 4A EU-TIRADS, K-TIRADS, and ACR-TIRADS considered as category 4. Excluded Bethesda I or non-informative material.

**Table 6:** Comparison between risk of malignancy (%) of L-TIRADS, EU-TIRADS, K-TIRADS, and ACR-TIRADS categories.

Categories	Based on L-TIRADS	Based on EU-TIRADS	Based on K-TIRADS	Based on ARC-TIRADS
Risk of malignancy of TIRADS categories (%)				
TIRADS1	-	-	-	0
TIRADS 2	0	0	0	0
TIRADS 3	0.007	0.013	0.013	0.015
TIRADS 4A *	0.147	0	0.014	0.019
TIRADS 4B	0.28			
TIRADS 4C	0.41			
TIRADS 5	0.58	0.29	0.31	0.13

* 4A EU-TIRADS, K-TIRADS, and ACR-TIRADS considered as category 4.

**Table 7:** Pooled estimates of the sensitivity, specificity, PPV, NPV, accuracy, and area under curve (AUC).

TIRADS System	Included Values	SEN, %	SPE, %	PPV, %	NPV, %	ACC, %	95% CI	AUC	*p*-Value
L-TIRADS	TIRADS 4A–5	80.6	72.7	29.6	96.3	73.3	0.68–0.84	0.766	<0.0001
EU-TIRADS	TIRADS 4–5	97.2	39.9	18.7	99.0	47.0	0.61–0.76	0.686	<0.0001
K-TIRADS	TIRADS 4–5	97.2	46.6	20.6	99.2	53.9	0.65–0.79	0.719	<0.0001
ACR-TIRADS	TIRADS 4–5	97.2	41.1	19.0	99.0	48.0	0.61–0.76	0.692	<0.0001

**Table 8:** Comparison between the L -TIRADS and EU-TIRADS classifications.

L-TIRADS		EU-TIRADS			Total
	2	3	4	5	
2	1	1	0	0	2
3	3	74	74	4	155
4A	0	2	8	57	67
4B	0	0	0	29	29
4C	0	0	0	23	23
5	0	0	0	13	13
Total	4	77	82	126	289

**Table 9:** Comparison between the L -TIRADS and K-TIRADS classifications.

L TIRADS	K-TIRADS				Total
	2	3	4	5	
2	1	1	0	0	2
3	15	81	56	3	155
4A	2	0	17	48	67
4B	0	0	0	29	29
4C	0	0	0	23	23
5	0	0	0	13	13
Total	18	82	73	113	289

**Table 10:** Comparison between the L -TIRADS and ACR-TIRADS classifications.

L TIRADS		ACR TIRADS				Total
	1	2	3	4	5	
2	1	0	1	0	0	2
3	2	5	69	76	3	155
4A	0	0	2	31	34	67
4B	0	0	0	1	28	29
4C	0	0	0	0	23	23
5	0	0	0	1	12	13
Total	3	5	71	108	103	287

## Data Availability

Data are available from the corresponding author after appropriative review.
